# Improved Subtyping of Avian Influenza Viruses Using an RT-qPCR-Based Low Density Array: ‘Riems Influenza a Typing Array’, Version 2 (RITA-2)

**DOI:** 10.3390/v14020415

**Published:** 2022-02-17

**Authors:** Kareem E. Hassan, Ann Kathrin Ahrens, Ahmed Ali, Magdy F. El-Kady, Hafez M. Hafez, Thomas C. Mettenleiter, Martin Beer, Timm Harder

**Affiliations:** 1 Institute of Diagnostic Virology, Friedrich-Loeffler-Institut, 17493 Greifswald-Insel Riems, Germany; kareem_eid@yahoo.com (K.E.H.); annkathrin.ahrens@fli.de (A.K.A.); martin.beer@fli.de (M.B.); 2 Department of Poultry Diseases, Faculty of Veterinary Medicine, Beni-Suef University, Beni-Suef 62511, Egypt; ali.178w@yahoo.com (A.A.); mfelkady@yahoo.com (M.F.E.-K.); 3 Institute of Poultry Diseases, Free University Berlin, 14163 Berlin, Germany; hafez.mohamed@fu-berlin.de; 4 Friedrich-Loeffler-Institute, 17493 Greifswald-Insel Riems, Germany; thomas.mettenleiter@fli.de

**Keywords:** avian influenza, diagnosis, real-time RT-PCR, Newcastle disease virus, infectious bronchitis virus

## Abstract

Avian influenza virus (AIV) variants emerge frequently, which challenges rapid diagnosis. Appropriate diagnosis reaching the sub- and pathotype level is the basis of combatting notifiable AIV infections. Real-time RT-PCR (RT-qPCR) has become a standard diagnostic tool. Here, a total of 24 arrayed RT-qPCRs is introduced for full subtyping of 16 hemagglutinin and nine neuraminidase subtypes of AIV. This array, designated Riems Influenza A Typing Array version 2 (RITA-2), represents an updated and economized version of the RITA-1 array previously published by Hoffmann et al. RITA-2 provides improved integration of assays (24 instead of 32 parallel reactions) and reduced assay volume (12.5 µL). The technique also adds RT-qPCRs to detect Newcastle Disease (NDV) and Infectious Bronchitis viruses (IBV). In addition, it maximizes inclusivity (all sequences within one subtype) and exclusivity (no intersubtypic cross-reactions) as shown in validation runs using a panel of 428 AIV reference isolates, 15 reference samples each of NDV and IBV, and 122 clinical samples. The open format of RITA-2 is particularly tailored to subtyping influenza A virus of avian hosts and Eurasian geographic origin. Decoupling and re-arranging selected RT-qPCRs to detect specific AIV variants causing epizootic outbreaks with a temporal and/or geographic restriction is possible.

## 1. Introduction

Highly pathogenic avian influenza virus (HPAIV) is the major pathogen associated with substantial economic losses in poultry production. Zoonotic AIV strains, in addition, have caused multiple cases of human infections, sparking influenza pandemic concerns [1]. The influenza A virus genome consists of eight single-stranded RNA gene segments. Two segments encode the major envelope glycoproteins species of these viruses, hemagglutinin (HA) and neuraminidase (NA). These proteins have essential functions in defining host and tissue tropism and influence virulence. The HA, in particular, is a main target of the protective humoral immune response. Based on nucleotide sequence and protein antigenicity of the HA and NA surface glycoproteins, AIVs are classified into 16 different HA (H1–H16) and 9 NA subtypes (N1–N9) [2,3]. The distinctive segmental structure of influenza virus genomes enables reassortment of segments if the same host cell is infected by two (or more) different parental viruses. Theoretically, 144 combinations between HA and NA may result. However, not all of these have actually been detected in nature as there seem to be predilections of certain HA and NA combinations [4]. Moreover, there is a continuous turnover, with temporal and geographical restrictions, of different AIV subtypes and their variants in the reservoir hosts as in aquatic wild bird populations [5]. Spill-over infections into poultry populations, often starting endemic transmission chains with similar reassortment events, widen the replication basis of these viruses. Error-prone viral genome replication drives genetic drift and further increases genetic and antigenic variability [1,4]. In summary, these processes present a continuous challenge not only for the immune system of the avian hosts but also for accurate and rapid laboratory diagnosis. 

Swift and exact diagnosis of AIV infections in poultry populations is pivotal to inform veterinary authorities and steer restriction measures if notifiable AIV subtypes, i.e., H5 and H7, are detected. Adequate control of zoonotic AIV infections in poultry populations is also the most important measure to limit exposure of human populations to these viruses [6]. Aquatic wild birds play a major role in the evolution, maintenance, and spread of AIV. Therefore, optimized surveillance of reservoir populations is important to follow viral evolutionary trajectories [7]. Conventional techniques for AIV diagnosis include virus isolation in embryonated chicken eggs, serological characterization of virus isolates, and animal experiments to define viral pathogenicity [4]. Time until final diagnosis using these methods may take up to two weeks. Minimizing time until diagnosis, therefore, is the main objective of new diagnostic developments. Rapid antigen detection assays based on lateral flow immunochromatography were particularly successful in this respect but lacked sensitivity [8,9,10] when compared to reverse transcriptase-polymerase chain reaction (RT-PCR) and especially time-saving real-time RT-PCR technologies (RT-qPCR). Thus, RT-qPCRs have become new standards [11,12,13,14,15]. These include RT-qPCRs for generic AIV detection, subtyping, and pathotyping where the latter are targeting the HA cleavage site [16,17,18]. 

In 2016, Hoffmann et al. [16] published an assay assembling several subtype-specific RT-qPCRs into a low-density PCR array, designated as the ‘Riems Influenza A Typing Array’ (RITA). RITA enabled AIV RNA detection at subtype level in clinical samples by providing a generic, internally controlled M gene-specific duplex RT-qPCR and a further 31 monoplex TaqMan^®^-based RT-qPCRs to differentiate 14 HA and nine NA subtypes. Although RITA proved suitable for use in routine diagnostic applications, shortcomings in terms of minor cross-reactivities between closely related subtypes (e.g., H2/H5, H7/H10/H15, H1/H6) were noted. These effects caused subtle problems in ruling out co-infections with several AIV subtypes. In addition, the demand to performing in parallel 32 single RT-qPCRs might have a repelling effect on potential users.

In this study, an updated and improved version of RITA (syn. RITA-2) is developed and validated. The new version considers grossly enlarged databases of Eurasian AIV sequences since 2015, when the previous RITA version was designed. Intersubtypic cross-reactions have been abrogated by re-designing primers and probes so as to select target locations that were less conserved between the subtypes. Many primers and probes have been newly established so as to also meet the growing demands for full inclusivity of all Eurasian virus sequences of a certain subtype available in public databases. The revised version also achieved a higher degree of assay integration by using multiplexing of RT-qPCRs more stringently. Space on the array was economized to accommodate four instead of three clinical samples per 96-well array plate and to include all 16 HA and nine NA AIV subtypes in addition to an influenza A generic RT-qPCR. Also, targets for other important avian pathogens, Newcastle Disease virus (NDV) and Infectious Bronchitis virus (IBV), have been added to the design for differential diagnostic means. In addition, we show how the RITA-2 array can be used as an “assay mine” and single reactions be decoupled and recombined into much smaller arrays tailored to routine diagnosis during HPAIV epizootics.

## 2. Materials and Methods

### 2.1. Viruses

RNA extracted from 428 influenza viral strains representing 16 HA and nine NA subtypes pre-typed (either serologically or by sequencing) at the National Reference Laboratory for Avian Influenza, Friedrich-Loeffler-Institut (NRL-AI, FLI, Isle of Riems, Germany) were used for analytical validation of the newly developed assays. Table 1 provides a condensed overview of the subtypes used and their species of origin. In addition, fifteen reference isolates each for IB and ND viruses were used for re-evaluation of previously established IBV and NDV RT-qPCR assays (Table S1).

### 2.2. Clinical Material

Oropharyngeal and cloacal swabs collected from diseased or freshly dead birds were submitted to the NRL-AI. Egyptian samples consisted of pools of 15 to 20 swabs from each flock of poultry and were collected from duck, chicken, and turkey farms in Egypt during 2018 and from Egyptian chicken flocks during early 2019 (47 samples in total). Samples were suspended in 2 mL of phosphate-buffered saline (PBS), pH 7.0–7.4, and clarified by centrifugation at 4000 rpm for 10 min at the Department of Poultry Diseases (Beni-Suef University, Beni Suef, Egypt). About 200 μL of clarified supernatant were uploaded on Whatman^®^ FTA^®^ Cards (Merck, Darmstadt, Germany) and sent to NRL-AI at FLI, Germany. In addition, oro-nasal and cloacal swabs collected from clinically healthy duck flocks from Bangladesh during 2019 were included (13 samples in total) as well as routine diagnostic samples submitted for diagnosis during HPAI epizootics in Germany (*n* = 62; Table S2).

### 2.3. Primers and Probes

Primers and probes reported for version 1 of the RITA array were double-checked against the EpiFlu database of GISAID (http://platform.gisaid.org), the International Nucleotide Sequence Data Collaboration IRD (http://www.fludb.org) sequence databases or against comprehensive alignments of HA and NA sequences built from these. Sequences were handled using the Geneious software, version 11.1.7 [19]. Alignment and identity matrix analyses were generated using MAFFT [20] and manually edited with AliView [21]. The focus of searches and comparisons was on sequences added to the databases since 2015. The chemical properties of preselected oligos were analyzed by the OligoCalc software [22]. Oligos were synthesized by Metabion GmbH (Martinsried, Germany) and Eurogentec (Liège, Belgium). Oligos were solubilized to produce stock solutions of 100 pmol µL^−1^ and stored at −20 °C until use. For use in PCRs, stock solutions were diluted to give a final concentration of 5–20 pmol µL^−1^ depending on individual reactions as shown in Table 2. 

**Table 2:** The final design of primers and probes used for assembling the RITA-2 array.

Subtype	Designation	Sequence 5’⇒3’	Amount ^1^	Reference
**Pan AI assay**	**M1-F**	AGA TGA GYC TTC TAA CCG AGG TCG	20.0 µL	[4,15]
	**M1-FAM**	FAM-TCA GGC CCC CTC AAA GCC GA-BHQ1	2.5 µL	
	**M1-R1**	TGC AAA AAC ATC TTC AAG TYT CTG	15.0 µL	
	**M1-R2**	TGC AAA GAC ACT TTC CAG TCT CTG	15.0 µL	
	**M1-R3**	TGC AAA I(Inosine)AC ATC YTC AAG TYT CTG	7 µL	
**H1 assay**	**H1-F1**	CCA TCT GTA TAG GCT AYC AT	20 µL	This study ^2^
	**H1-F2**	AAA CAT YCC TTC CRT TCA ATC	20 µL	
	**H1-FAM1**	FAM-TAC AGA CAC TGT YGA CAC DGT GCT-BHQ1	5 µL	
	**H1-FAM2**	FAM-TTC ATT GAA GGR GGR TGG ACA GGA AT-BHQ1	5 µL	
	**H1-R1**	GTG AGT CAC RGT YAC ATT CTT	20 µL	
	**H1-R2**	GAG CAA GGI TCY GGT TAT G	20 µL	
**H2 assay**	**H2-F**	CTA AST GTR CCW GAA TGG TC	40 µL	This study ^2^
	**H2-R**	GAG GTG TTT CAR TTC YTC RTA	40 µL	
	**H2-FAM**	FAM-TGT GCT ACC CAG GYA GTT TCA ATG A -BHQ1	8 µL	
**H3 assay**	**H3-F1**	CCT CGR GGC TAY TTC AAR AT	15 µL	This study ^2^
	**H3-F2**	AGA CTG GAT CYT RTG GAT TTC	15 µL	
	**H3-F3**	CTG GGR CAC CAT GCA GT	15 µL	
	**H3-FAM1**	FAM-TGC ATC TGA YCT CAT TAT YGA RCT TTT-BHQ1	4 µL	
	**H3-FAM2**	FAM-ACR CAA AGC AAA AAG CAT GAT ATG GC-BHQ1	4 µL	
	**H3-FAM3**	FAM-ACA GGG AAA ATA TGC ARC AAT CCY CA-BHQ1	4 µL	
	**H3-R1**	ATT TGG RGT GAT RCA TTC AGA	15 µL	
	**H3-R2**	CTC AAA TGC AAA TGK TGC AYC	15 µL	
	**H3-R3**	TGT GCA GTC YCT TCC ATC	15 µL	
**H4 assay**	**H4-F1**	ACYCAGGGRTACAAGGACA	20 µL	This study ^2^
	**H4-F2**	GGA CAT CAT YCT YTG GAT TTC	20 µL	
	**H4-FAM**	FAM-TCC ATA TCA TGC TTY TTG CTY GTA GC-BHQ1	4 µL	
	**H4-R**	CAA GCC CAC AAA AYR AAG G	40 µL	
**H5 assay**	**H5-HA1-F**	GAT TYT AAA RGA TTG TAG YGT AGC	20 µL	This study ^2^
	**H5-FAM3-RC**	FAM-CGC ACA TTG GRT TYC CRA GGA GCC-BHQ1	6 µL	
	**H5-HA1-R1**	CTC TCY ACC ATG TAR GAC CA	15 µL	
	**H5-HA1-R2**	CTC TCY ACT ATG TAR GAC CA	15 µL	
	**H5-F2**	GTT CCC TAG YAY TGG CAA TCA T	20 µL	
	**H5-FAM2**	FAM-CTG GTC TAT YYT TRT GGA TGT GCT CC-BHQ1	6 µL	
	**H5-R2**	AAT TCT ARA TGC AAA TTC TGC AYT G	15 µL	
**H6 assay**	**H6-F1**	TTG GYG TGT ATC AAA TYC TTK C	20 µL	This study ^2^
	**H6-F2**	TTG RCG TGT ATC AAA TAC TTG C	20 µL	
	**H6-FAM**	FAM-AGR CTG CTC GAY ACC GTA CTA TAA A-BHQ1	10 µL	
	**H6-R**	TTGA RCY ATT TGA ACA CAT CCA	40 µL	
**H7 assay**	**H7-F**	CAA CTG AAA CRG TRG ARC G	45 µL	This study ^2^
	**H7-FAM**	FAM-CCC AGG ATY TGC TCA ARA GGR AAA A-BHQ1	10 µL	
	**H7-R1**	CAG GAG YCC ACA TTG ACC	15 µL	
	**H7-R2**	CAG WAG YCC ACA TTG ACC	15 µL	
	**H7-R3**	TTC TAG GAA TTG GTC ACA TTG	15 µL	
**H8 assay**	**H8-F**	CCA CCT AYA AAA TTC TCA GCA	40 µL	This study ^2^
	**H8-FAM**	FAM-TGC CAA GCA RAG ACT GGC CGC CA-BHQ1	4 µL	[16]
	**H8-R**	ARA CCT CCA GCA AYC AGG A	40 µL	This study ^2^
**H9 assay**	**H9-F1**	CAA TGG GGT TYG CTG CCT	20 µL	[23]
	**H9-F2**	CAA TGG GRK TTG CTG CCT	20 µL	
	**H9-FAM**	FAM-TTY TGG GCC ATG TCI AAT GGR TC-BHQ1	6 µL	
	**H9-R**	TTA TAT ACA RAT GTT GCA YCT G	40 µL	
**H10 assay**	**H10-F**	CAA CTC AGR CAG AAT GCW GA	40 µL	This study ^2^
	**H10-FAM**	FAM-TGC ATG GAG AGY ATA AGR AAC AAC AC-BHQ1	6 µL	
	**H10-R**	CTT CYT CTC TGT AYT GTG AAT G	40 µL	
**H11 assay**	**H11-F**	GGA CAT ATG AYC ACA ARG AAT T	40 µL	This study ^2^
	**H11-FAM**	FAM-ACT GTC RAT TTA CAG CTG CAT YGC A-BHQ1	8 µL	
	**H11-R**	ATG CAA ATG GTA CAT CTA CAT G	40 µL	
**H12 assay**	**H12-F**	CAT CTA CAG CAG YGT YGC	40 µL	This study ^2^
	**H12-FAM**	FAM-ACT GCT CAT GAT TAT TGG GGG TTT CA-BHQ1	12 µL	
	**H12-R**	GAA AGT ACA ACG AAC ATT TCC A	40 µL	
**H13 assay**	**H13-F1**	CTT AAG CAC AAA CTC ATC AGA A	15 µL	This study ^2^
	**H13-F2**	CTG AGC ACC AAT TCA TCA GA	15 µL	
	**H13-F3**	CTT AAG CAC AAA CTC ATC AGA A	15 µL	
	**H13-FAM1**	FAM-CKA ACC ACA CRG GAA CAT AYT GTT C-BHQ1	5 µL	
	**H13-FAM2**	FAM-CAC ACI GGA ACA TWC TGT TCA ATC A-BHQ1	5 µL	
	**H13-R1**	CTG GCA CAG GCA GGG TT	20 µL	
	**H13-R2**	CCY ACA ATC CAT CCT TCA AA	20 µL	
**H14 assay**	**H14-F**	CCC AAT ATA GGA AGT AGA CC	40 µL	This study ^2^
	**H14-HEX**	HEX-AAG CAT CTA CTG GAC YCT AGT AAA CC-BHQ1	6 µL	
	**H14-R**	CTT CTT GTC ACT TYT AAG CAC	40 µL	
**H15 assay**	**H15-F**	CAS CTT TCT CCG CTC TAA TG	40 µL	This study ^2^
	**H15-FAM**	FAM-CAC TGG GAA TAC AGA GTG ATG CAC AA-BHQ1	3 µL	
	**H15-R**	AAR CAT TCC CCT TCA CAT GA	40 µL	
**H16 assay**	**H16-F**	ARY TGA AGA CTG AAG ACA ATG T	40 µL	This study ^2^
	**H16-HEX**	HEX-CTG GTA GGW CTC ATA CTY GCA TTT AT-BHQ1	6 µL	
	**H16-R**	CCA CTG CTG CAT GCC CA	40 µL	
**N1 assay**	**N1-F**	GRC CTT GYT TCT GGG TKG A	40 µL	This study ^2^
	**N1-FAM**	FAM-CAA TYT GGA CYA GTG GRA GYA GCA T-BHQ1	6 µL	
	**N1-R**	ACC GTC TGG CCA AGA CCA	40 µL	[16]
**N2 assay**	**N2-F1**	AGTC TGG TGG ACY TCA AAY AG	20 µL	[16]
	**N2-F2**	CAG AGT RTG GTG GAC ITC	20 µL	[23]
	**N2-FAM**	FAM-CAT CAG GCC ATG AGC CTG TYC CAT-BHQ1	4 µL	
	**N2-R**	TTG CGA AAG CTT AYA TNG VCA T	40 µL	
**N3 assay**	**N3-F**	GCA AYA GTA TAG TTA CYT TCT G	40 µL	This study ^2^
	**N3-FAM**	FAM-AGA CAA TGA ACC TGG ATC GGG VAA-BHQ1	3 µL	
	**N3-R1**	TTA CTT GGG CAT RAA CCC AAT	20 µL	
	**N3-R2**	GTT GGM ACC RTC WGG CCA	20 µL	
**N4 assay**	**N4-F1**	GAC TAG YGG TAG TAG YAT TGC	20 µL	This study ^2^
	**N4-F2**	AGT AGY ATT GCR TTY TGT GGT GTT	20 µL	[16]
	**N4-HEX**	HEX-TGG TCR TGG CCY GAT GGC GCT CT-BHQ1	6 µL	
	**N4-R**	CGA AAA ATY ACT TGT CTA TGT CAA	40 µL	This study ^2^
**N5 assay**	**N5-F1**	CCT TCA GAA TGC AGR ACY TT	20 µL	This study ^2^
	**N5-F2**	CAA ATA ATA CAG TAA ARG ACA GAA G	20 µL	
	**N5-HEX**	HEX-TAA TGA GCG TRC CAT TGG GAT CCT C-BHQ1	6 µL	
	**N5-RR**	TAG CAG ACC AYC CRA CGG A	40 µL	
**N6 assay**	**N6-F1**	GGT GAM AAT GAA YCC AAA YCA	15 µL	[16]
	**N6-F2**	AAT GAA YCC AAA YCA RAA GAT AA	15 µL	
	**N6-F3**	GAA AAT GAA TCC AAA TCA RAA GRT A	15 µL	This study ^2^
	**N6-FAM**	FAM-CAT YTC AGC IAG GAR TRA CAC TAT C-BHQ1	12 µL	
	**N6-R1**	CTT RTA RTG RAG TCC GAT GTT	15 µL	
	**N6-R2**	GAT TCC TAT YAG SAG GCT TAC	15 µL	
	**N6-R3**	GAT TCC TAT YAG SAI ICT TAC	15 µL	
**N7 assay**	**N7-F1**	GTT GAA TTA ATW AGA GGA AGR CC	20 µL	[16]
	**N7-F2**	AGA GGC YAA ATA YGT RTG GTG	20 µL	This study ^2^
	**N7-FAM**	FAM-CCT ATG TGG RAG CCC ATT CCC AGT-BHQ1	3 µL	
	**N7-R**	GA TYT GTG CCC CAT CRG GGA	40 µL	[16]
**N8 assay**	**N8-F1**	TCC ATG YTT TTG GGT TGA RAT GAT	15 µL	[16]
	**N8-F2**	CTG ATC TCT CTT ACA GGG TTG	15 µL	This study ^2^
	**N8-F3**	TCC ATG YTT TTG GGT IGA AAY GAT	15 µL	[16]
	**N8-FAM1**	FAM-TCH AGY AGC TCC ATT GTR ATG TGT GGA GT-BHQ1	6 µL	[16]
	**N8-FAM2**	FAM-TGC CCA GTG ACA CTC CAA GAG GGG AA-BHQ1	6 µL	This study ^2^
	**N8-R1**	GCT CCA TCR TGC CAY GAC CA	20 µL	[16]
	**N8-R2**	GTG CAT GAA CCG ACA AAT TGA G	20 µL	This study ^2^
**N9 assay**	**N9-F**	AGY ATA GTA TCR ATG TGT TCC AG	40 µL	[14]
	**N9-FAM**	FAM-TTC CTR GGA CAA TGG RAC TGG CC-BHQ1	3 µL	[16]
	**N9-R**	GTA CTC TAT TYT AGC CCC RTC	40 µL	This study ^2^
**NDV assay**	**NDF**	GAG CTA ATG AAC ATT CTT TC	12.5 µL	[23]
	**NDR**	AAT AGG CGG ACC ACA TCT G	12.5 µL	
	**ND-FAM1**	FAM-TCA TTC TTT ATA GAG GTA TCT TCA TCA TA-BHQ1	4 µL	
	**ND-FAM2**	FAM-TCA TAC ACT ATT ATG GCG TCA TTC TT-BHQ1	4 µL	
**IBV assay**	**IBV-F1**	CAG TCC CDG ATG CNT GGT A	25 µL	[24]
	**IBV-F2**	CAG TCC CDG ACG CGT GGT A	25 µL	
	**IBV-F3**	GCT TTT GAG CCT AGC GTT	5 µL	
	**IBV-FAM1**	FAM-ACT GGA ACA GGA CCD GCC GCT GAC CT-BHQ1	6 µL	
	**IBV-FAM2**	FAM-CAC CAC CAG AAC CTG TCA CCT C-BHQ1	2 µL	
	**IBV-R1**	CCT TWS CAG MAA CMC ACA CT	25 µL	
	**IBV-R2**	GCC ATG TTG TCA CTG TCT ATT G	5 µL	
**IC-2**	**EGFP-1-F**	GAC CAC TAC CAG CAG AAC AC	5 µL	[25]
	**EGFP-10-R**	CTT GTA CAG CTC GTC CAT GC	5 µL	
	**EGFP-HEX**	HEX-AGC ACC CAG TCC GCC CTG AGC A-BHQ1	3.75 µL	

^1^ A stock mix of 200 µL was produced for each assay; the amount in µL of a 100 pmol µL^−1^ solution of each primer and probe for the stock mix is given here. 0.1 × TE buffer was then added up to a final volume of 200 µL. Finally, 1 µL of the stock mix was used per PCR reaction. ^2^ Positions shown in red have changed in comparison to the RITA-1 array. Oligonucleotides shown completely in red have been newly designed in this study. IC—Internal control system based on an RNA run-off transcript of a fragment of the EGFP gene [25].

### 2.4. RNA Extraction

Viral RNA was extracted from infected MDCK cell cultures supernatants or allantoic fluids of embryonated chicken eggs using the NucleoMag^®^VET Kit (Macherey-Nagel GmbH & Co. KG, Düren, Germany) according to the manufacturer’s instructions. Clinical material (swab samples) was extracted manually using the Qiagen Viral RNA kit (Qiagen, Hilden, Germany) or by the Qiagen MagAttract Kit operated on a KingFisher Biosprint96 device (Qiagen). Samples sent on FTA cards were extracted as described by [26], using the QIAamp Viral RNA Mini Kit (Qiagen). Nucleic acids were eluted in 70 μL of nuclease-free water, and aliquots of 10 μL were stored at −20 °C until use. 

### 2.5. Plate Design 

**Figure 1:**
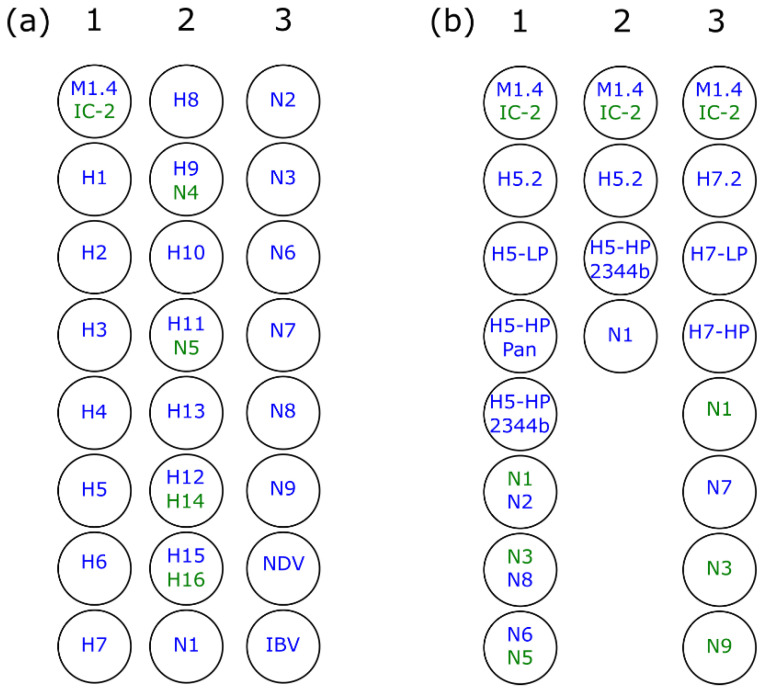
(**a**) 24-well plate layout (1–3) of the RITA-2 array allowing simultaneous testing of four clinical samples on a whole 96-well plate. Some reactions were decoupled from the RITA-2 format and newly recombined with additional reactions in eight- and four-well format for use in routine diagnostics tailored for epizootic outbreaks of notifiable AIV (**b**) 1- Eight-well layout for sub- and pathotyping of Eurasian H5 viruses, 2- Four-well layout for sub- and pathotyping of viruses encountered during the current (autumn 2021) HPAIV H5N1 epizootic, 3- Eight well layout for sub- and pathotyping of Eurasian H7 viruses. Subtype color indicates the type of reporter dye, blue—FAM, green—HEX.

The layout of the RITA-2 array aimed at economizing space by integrating several targets into duplex PCR reactions. As depicted in Figure 1a, the layout allows the testing of four samples simultaneously on one plate. Batches of plates ready-for-use were prepared and stored at −20 °C by pipetting 1 µL of primer-probe stock mixes into the fixed positions as shown in Figure 1a before freezing.

In the same way, strips of eight or four wells were prepared with single PCR reactions decoupled from the RITA-2 design and recombined with previously published pathotyping RT-qPCRs H5-LP, H5-HP Pan, H5-HP 2.3.4.4 [17] or H7-LP and H7-HP [18] (Figure 1b).

### 2.6. Set-Up of RT-qPCR Reactions 

RT-qPCRs were run on a Bio-Rad CFX 96 real-time PCR machine (Bio-Rad, Munich, Germany) in the 96-well format using the AgPath-ID One-Step kit (Applied Biosystems, Foster City, CA, USA) and non-skirted, low profile, white qPCR 96-well plates (RT-PL96-OPWSA, Eurogentec, Liège, Belgium). For 8- and 4-well designs (Figure 1b) also 0.2 mL 8-Tube PCR Strips (low profile, white #TLS0851, Bio-Rad, Munich, Germany) were used. A heterologous internal control system (IC-2) was used to check the performance of reverse transcription and PCR amplification [26]. Details of the reaction mix set-up are shown in Table 3, whereas × should be understood as multiplication sign. Primer-probe stock mixes had already been pipetted into plates at fixed positions, as shown in Figure 1. Fully prepared plates were sealed and kept frozen at −20 °C until use. Plates were prepared in batches of 20–40 plates. The total PCR volume of a single reaction comprised 12.5 µL to which 2.5 µL of extracted RNA was added. CFX96 machines were programmed as follows: Reverse transcription 45 °C for 10 min, initial denaturation 95 °C for 10 min, 45 cycles of denaturation 95 °C for 15 s, annealing/reading (of FAM and HEX channels) 56.5 °C for 20 s, and elongation 72 °C for 30 s. The Cq threshold was set to <40.

### 2.7. Preparation of Positive Controls

Different plate batches were evaluated by a set of four positive controls, each consisting of RNA of five different viruses, as shown in Table 4. RNA of individual viruses was mixed in RNA safe buffer [16] (0.05% *v*/*v* Tween 20, 0.05% *w*/*v* sodium azide, 50 ng μL^−1^ of carrier RNA [poly(A) homopolymer; Amersham Biosciences, Piscataway, NJ, USA]) to ensure a Cq value of 28–32 in the M-PCR. PTCs were frozen at −80 °C until use. The identification of strains used to compile the PTCs is given in Supplementary Materials Table S2. Each batch of plates was also checked with a no-template control.

### 2.8. Statistical Analyses 

Statistical analyses for sensitivity, specificity, intra-, and inter-assay variation were performed using the SigmaPlot software, version 11 (Systat Software Inc., Duesseldorf, Germany). Spearman’s rank correlation and Student’s t-test were employed. *p* < 0.05 was considered significant.

## 3. Results

### 3.1. Evaluation and Selection of Oligonucleotide Sets 

In silico analyses of comprehensive sequence alignments for each HA and NA subtype of Eurasian origin revealed, for the majority of subtypes, a within-subtype variation that was too wide to be covered by a single set of primers and probe; inclusion of sequences of American or Australian origin grossly increased such variation. Therefore, it was decided, as a continuation of the array strategy of RITA-1, to restrict oligonucleotide selection to Eurasian viruses. In order to avoid a large number of degenerate positions, two (e.g., H1, H5, N8) and even three separate sets (H3) of primers and probes had to be designed to cover the full width of sequence variation of these subtypes (Table 2). The PCRs were du- or triplexed within each subtype and probes specific for the same subtype labeled with the same reporter dye. As indicated by the red color of nucleotides in Table 2, the majority of oligonucleotides used in RITA-1 had to be modified or fully replaced to qualify for inclusion into RITA-2. Primer and probe sets were tested extensively against selected reference isolates of the matching and closely related subtypes with several rounds of optimization. The final selection was then successfully tested against all homosubtypic isolates as listed in Table 1. 

### 3.2. Extended Target Spectrum of RITA-2

To economize space on the final PCR array, RT-qPCRs specific for several different subtypes were duplexed using FAM and HEX reporter dyes (Figure 1a: M/IC2; H9/N4; H11/N5; H12/H14; H15/H16); care was taken to combine subtypes that have not been detected so far in nature to avoid competition during amplification. 

**Figure 2:**
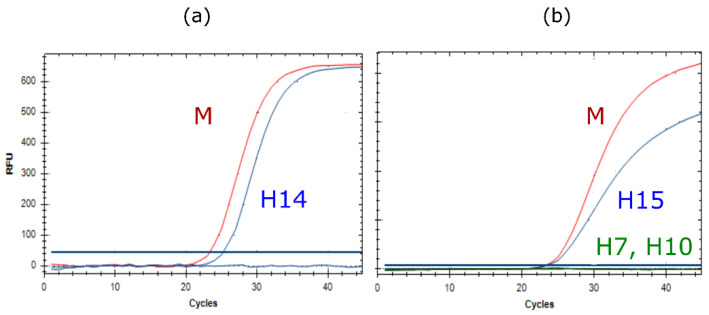
RT-qPCRs specific for avian influenza virus subtypes (**a**) H14 (A/Mallard/Gurjev/263/82), and (**b**) H15 (A/Shearwater/West Australia/2576/79), in comparison to influenza A virus-generic M-PCR (red, in (**a**,**b**)). Subtypes H7 and H10 (green in (**b**)), closely related to H15 did not cross-react.

While RITA-1 provided no reactions to identify subtypes H14 and H15, these have now been added to RITA-2. Figure 2 shows that these assays have a sensitivity comparable to the generic M-PCR. In addition, the H15 assay did not cross-react to the closely related subtypes H7 and H10. Unfortunately, only a single reference isolate was available each for AIV subtypes H14 and H15. 

According to recent data from Egypt, poultry flocks showing respiratory disease and increased mortality were often found to suffer from co-infections of AIV with NDV and/or IBV [23,24]. Likewise, viral isolates were found to harbor a mix of AIV, NDV, and/or IBV [27,28,29]. In addition, NDV and IBV infections are among the most important differential diagnoses of HPAI in poultry. Therefore, in RITA-2, NDV- and IBV-specific RT-qPCRs have been included. Rather than designing new assays, approved published methods have been adopted here (Table 2).

**Figure 3:**
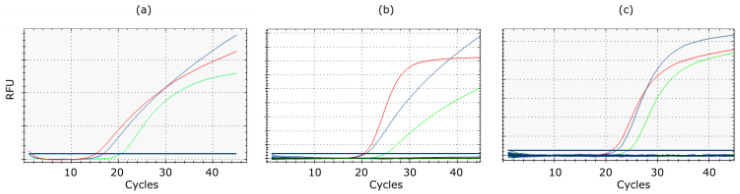
Improved sensitivity of RT-qPCRs specific for avian influenza virus subtypes (**a**) H3 (A/Mallard/Germany/R1648/07 [H3N8]), (**b**) N2 (A/Chicken/Egypt/AR538/2017 [H5N2 hp]), and (**c**) N4 (A/Mallard /Germany/R2167/2009 [H8N4]) in RITA-2 (blue) compared to RITA-1 (green). Generic M-specific amplification curves are shown in red.

Routine use of RITA-1 for subtyping clinical samples isolates revealed a lower sensitivity of several subtype-specific assays compared to the generic M-PCR. Examples are shown in Figure 3. Re-designing primers and/or probes or selecting completely new target regions re-established sensitivity of the RITA-2 assays to the level of the M-PCR.

### 3.3. Analytical Sensitivity

**Figure 4:**
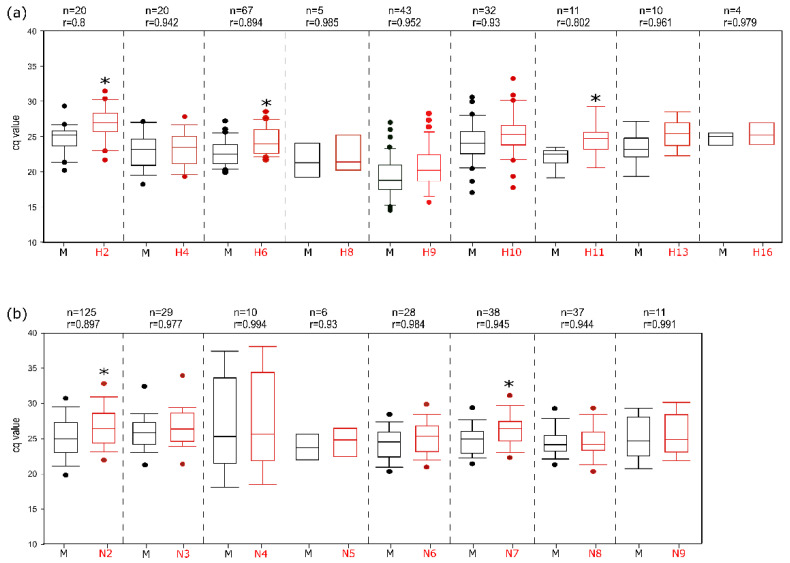
Subtype-specific analytical sensitivity of the RITA-2 array compared to the generic M-RTqPCR assay. (**a**) HA subtypes 2, 4, 6, 8, 9, 10, 11, 13, and 16; for subtypes H14 and H15, only a single isolate was available (s. Figure 2). (**b**) NA subtypes 2–9. *n*—Number of isolates tested, r—Spearman’s rank correlation coefficient, *—statistically significant difference between the Cq values of the generic and the subtype-specific RT-qPCRs. Dots define outliers.

**Figure 5:**
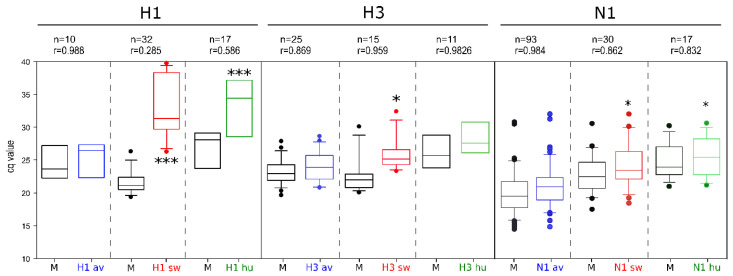
Influence of host origin of virus isolates on the analytical sensitivity of the RITA-2 array. *n*—Number of isolates tested, r—Spearman’s rank correlation coefficient, *—Statistically significant difference. ***—Highly significant difference; av, sw, hu—avian (blue), swine (red), human (green) host origin. Dots define outliers.

All subtype-specific assays were evaluated with all the available matched subtypes and compared to the generic M-PCR. A wide range of isolates regarding time, place of origin, and host species was used. The analyses showed that most of the subtype-specific assays attained the level of sensitivity of the M-PCR as indicated by correlation coefficients > 0.93 (Figure 4). For some assays (H2, H6, H11, N2, N7), however, a statistically significant (*p* < 0.05) lower sensitivity of up to 3 Cq value on average was calculated. For H1, H3, and N1 subtypes, the host species origin was found to modulate sensitivity (Figure 5), and targets of non-avian origin were detected with significantly lower sensitivity due to mismatches in primers and/or probes. The H1 subtypes of human and swine-origin were particularly negatively affected. In order to address these findings in routine diagnostic settings, clinical samples with a viral load of Cq > 35 (as measured by a generic influenza A virus RT-qPCR) were not assigned to examination in the RITA-2 array.

**Figure 6:**
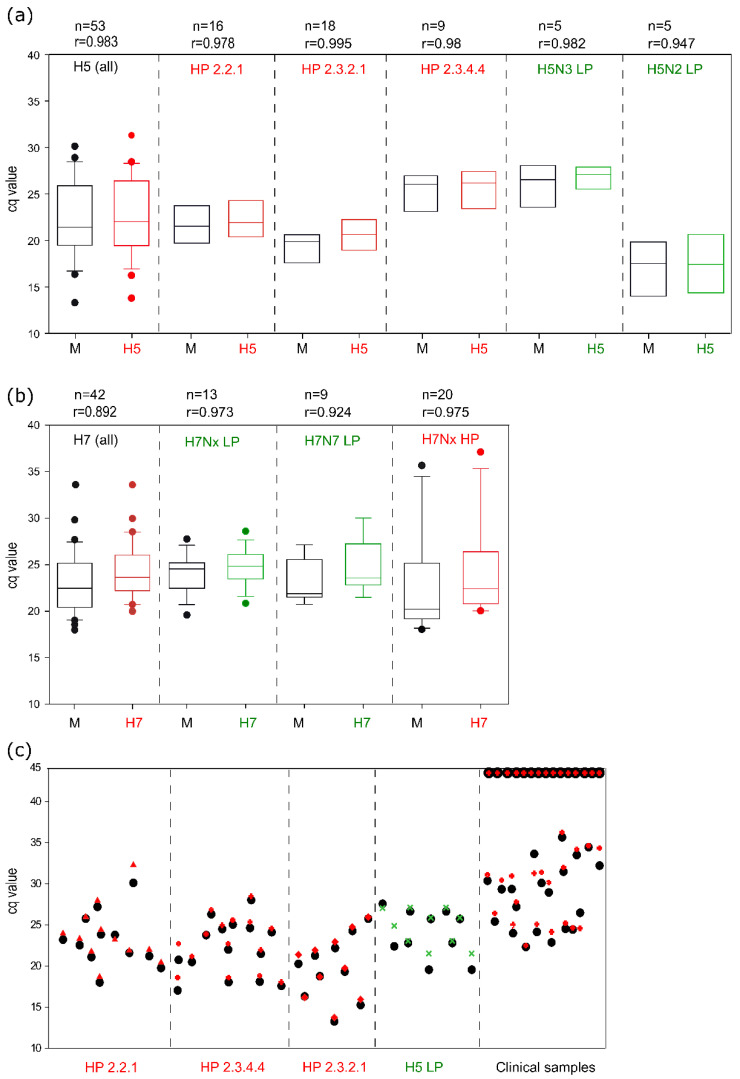
Analytical sensitivity of the RITA-2 array for notifiable avian influenza viruses of HA subtypes H5 (**a**) and H7 (**b**), stratified by phylogenetic lineage and pathotype (HP—red, LP—green) and (**c**) pairwise comparison of Cq values for individual isolates or clinical samples (including H5-negative ones) obtained by generic M-PCR (black dot) and the H5 subtype-specific RITA-2 assay (colored symbols). *n*—number of tested isolates, r—Spearman’s rank correlation coefficient.

Notifiable AIV of subtypes H5 and H7 received specific dedication (Figure 6): RITA-2 assays detected RNA of these subtypes with high sensitivity, independently of the pathotype and the clade of H5 viruses of the goose/Guangdong lineage.

### 3.4. Analytical Specificity

In RITA-1, several assays revealed minor intersubtypic cross-reactions, particularly between subtypes H1/H6, H2/H5, and H7/H10/H15 which are known to be genetically closely related. Therefore, special care was taken to re-design primers and probes to avoid such cross-reactions. Based on published phylogenetic panoramas for the HA and NA subtypes [30,31], a selected pattern of closely related subtypes was used to validate analytical specificity. 

**Figure 7:**
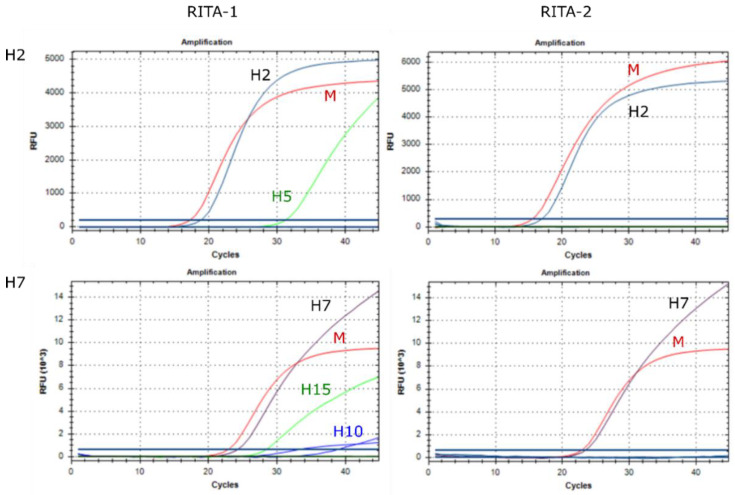
Intersubtypic cross-reactions of RITA-1 are resolved in re-designed RITA-2 assays. Here the following strains were used: H2 (A/Mallard/Germany/Wv677/04 [H2N3]), H5 (A/White stork/Germany/AR251/2018 [H5N6 hp]), H7 (A/Greylag goose/Germany/AR942/2015 [H7N7]), H10 (A/Mallard/Germany/1490/09 [H10N7]) and H15 (A/Shearwater/West Australia/2576/79 [H15N9]).

As shown in Figure 7, upper left panel, RITA-1 produced a highly specific signal for H2 when tested with an H2 isolate but also an H5 virus gave a (significantly weaker) positive signal in the H2 assay; the same was seen vice versa for the H5 assay. The problem was even more complex for H7 viruses, which are closely related to subtypes H10 and H15: In RITA-1, an H7 virus gave a highly specific signal in the H7 assay, but also H10 and H15 viruses tested (false-) positive in the H7 assay (Figure 7, lower left panel). Similarly, an H10 virus produced a weakly positive specific signal in the H7 assay. In RITA-2, these cross-reactions no longer exist (Figure 7, right panels). Similar results were obtained for all assays tested against the pattern of closely related subtypes. Thus, the RITA-2 array has increased in specificity.

### 3.5. Assay Robustness 

Duplicate runs of selected reference samples for all 16 HA and nine NA assays plus IBV and NDV targets on the same plate were used to evaluate intraassay variation. Inter-assay variation was investigated using two different thermal cyclers and two different plates for the same selected samples on two different days. As shown in Tables S3 and S4, the standard deviations and covariances calculated suggest excellent assay robustness.

### 3.6. Performance Characteristics with Clinical Samples

**Figure 8:**
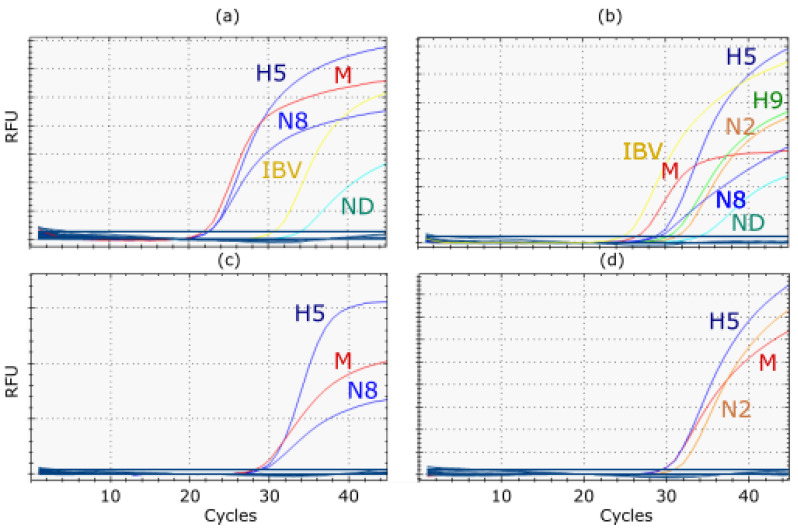
Examples of RITA-2 analysis of clinical (cloacal swab) samples from poultry flocks in Egypt detecting mixed infections of several AIV subtypes (**a**) Mixed infection with AIV H5N8, IBV and NDV; (**b**) Mixed infection with AIV H5, H9, N2, N8 IBV and NDV; (**c**) Mono-infection with AIV H5N8; (**d**) Mono-infection with AIV H5N2.

The diagnostic performance of RITA-2 was evaluated with a set of 60 clinical samples from poultry originating from Egypt and Bangladesh. RITA-2 succeeded in differentiating subtypes and detecting IBV and/or NDV with high sensitivity and specificity, as shown in Table 5. In RITA-2, all subtypes specified for these samples by other RT-PCRs and/or sequence analysis were confirmed (H5, H9, N1, N8, and N2). Some of these samples were also tested by RITA-1 but failed to detect the H9 subtype at lower viral loads. RITA-2 takes advantage of a set of primers and probes that we updated previously [23], which enabled detection of recent H9N2 viruses of the G1 lineage circulating in northern Africa with much higher sensitivity than an older H9 protocol [32]. Also, mixed infections of different AIV subtypes were detected; these results extended to co-infections with IBV and NDV in two samples, as depicted in Figure 8. 

In addition, we decoupled single assays from the RITA-2 array and re-arranged smaller arrays of eight or four wells (Figure 1b) comprising RT-qPCRs for: (i) generic AIV detection, (ii) H5 or H7 sub- and pathotyping, and (iii) fitting NA subtyping. This step was taken to guarantee high throughput of a demanding daily sample size during HPAI epizootics experienced in Europe 2016/17 and 2020/21. We analyzed a total of 63 clinical samples from the recent 2020/21 HPAI H5 epizootic in Europe (Supplementary Materials Table S3) and show that careful selection of single assays decoupled from RITA-2 allowed a full diagnosis regarding H5 sub- and pathotype as well as (in most cases) the NA subtype with a grossly reduced turn-around time as compared to the full RITA-2 or single RT-qPCRs and nucleotide sequencing for pathotyping. 

## 4. Discussion

Diagnostic tools for avian influenza viruses (AIV) are constantly challenged due to reassortment and genetic drift of these viruses. Recently, RT-qPCRs have been established as standards for rapid and sensitive diagnosis [4]. Due to the rapid evolutionary diversification of the AIV sequence cloud, particularly affecting the HA segment, subtyping RT-qPCRs are under exceptionally high pressure to adapt to the fluctuations of such clouds. Ideally, assays should be inclusive for all sequences published for a single subtype and, at the same time, exclusive for all sequences not clustering with this subtype. Suitably conserved but subtype-specific target sequences in the HA and NA genome segments become limited in reciprocal relation to the growing number of sequences in databases, as our in-silico study of comprehensive alignment sets has revealed. 

To re-establish high sensitivity and specificity of arrayed influenza virus subtype-specific RT-qPCRs for recently circulating viruses, we re-designed primers and probes previously published by Hoffmann et al. [16] and re-assembled those PCRs into an economized PCR array, termed RITA-2. An acceptable balance between a highly sensitive broad reactivity and full subtype specificity was finally achieved using multiplexed Taqman^®^ based technology. The use of two fluorescent marker dyes allowed integration of PCRs and reduced the set of previously 32 subtyping PCRs of RITA-1 to 24 in RITA-2. In addition, two further AIV subtypes (H14, H15) and targets for NDV and IBV, two important differential diagnoses of AIV infections in poultry, were accommodated into the array. The assay provides considerable versatility and robustness as all 24 wells for a single sample can be pipetted using a multichannel pipette and a single master mix per sample. The plates can be prepared in advance in batches with primers and probes pipetted into the correct positions and stored at −20 °C. A storage time of nine months in our hands did not lead to a loss of sensitivity. Cutting the plates if the full range of four samples per plate is not required can be accomplished as well. Reducing the total volume of the RT-qPCR reactions from 25 µL (RITA-1) to 12.5 µL per reaction further adds to cost reduction in RITA-2.

A continuing emergence of AIV variants characterized the evolution of highly pathogenic (HP) AIV of the Chinese Gs/GD lineage over the last two decades. NA subtype switching and an accelerated diversification of HA sequences leading to an intricate fragmentation into various phylogenetic clades, subclades, and lineages have been a hallmark of these viruses [33]. Appropriate diagnostic tools should be able to detect such diverse viruses, and the H5 assay implemented in RITA-2 achieves this goal as demonstrated by detecting representative HPAI H5 viruses from at least four recent clades (Figure 6a). Severe epizootic outbreaks of Gs/GD HP H5 viruses have been witnessed in Europe during the winter seasons of 2016/17 (mainly H5N8), 2020/21 (mainly H5N8, but also N1, N3, N4, and N5), and 2021/22 (mainly H5N1) [34,35]. Single subtyping assays of RITA-2 and pathotyping RT-qPCR published by Naguib et al. and Graaf et al. [17,18] were customized and re-arranged into 8- and 4-well arrays (Figure 1b, Table S2). In cases such as the mentioned HPAI epizootics, when most AIV-positive samples are expected to be dominated by a certain sub- and pathotype, smaller arrays can be used to save time and costs to establish a final diagnosis (Table S2).

Mixed infections with different influenza A virus subtypes are a prerequisite of reassortment. In addition, co-infections of AIV and other viral avian pathogens with clinical impact have recently been found at increasing frequency in poultry in countries like Egypt [23,24,27,28,29] and Bangladesh [36]. In Egypt, such co-infection gave rise to a diffuse clinical picture, termed “respiratory disease complex” in gallinaceous poultry [27]. We show here that RITA-2 detects mixed infections in clinical samples even if these targets are present in grossly different concentrations (Figure 8). Separating the amplification reaction into distinct wells obviates competition effects which would decrease the sensitivity of detecting minor targets. To detect mixed infections, it is essential to understand better the viral infection dynamics in individual birds and populations. Reassortment events “*in statu nascendi*” can be followed using RITA-2, and has led previously to the detection of a new HPAIV subtype, H5N2, in Egypt [11]. Sensitive virus isolation techniques would essentially cover at least the same range of viruses (AIV, NDV, IBV), but often certain viruses outcompete others in such mixtures [28,29]. This would lead to a skewed impression of the actual co-infections. Next-generation sequencing focusing on a true metagenomics approach would be adequate to uncover the full range of co-infecting pathogens in a sample [34]. However, such technology is still costly and less suitable than RT-qPCR for routine diagnostic laboratories. The capabilities of RITA-2 to detect mixed infection might also be useful when analyzing the purity of virus strains based on clinical isolates that are used as diagnostic antigens in serological assays or for (autologous) vaccine production.

Emerging new subtypes and drift variants of influenza A viruses pose an ultimate challenge to their diagnostic detection by sequence-based techniques such as RT-qPCR. There is extensive natural sequence variation between AIV subtypes circulating in wild birds and poultry in the Americas and Oceania compared to Eurasia [30,31]. In silico analyses of the available sequence information did not allow the selection of a global “one-fits-all” type of RT-qPCR for all the different subtypes. Therefore, like RITA-1, the new version-2 remains restricted to the analysis of viruses and samples obtained from wild birds and poultry of Eurasia and Africa. Nevertheless, it should be possible to design a similar array for the corresponding American or Oceanian lineages. Sequence variation was also related to the host origin of the viruses and negatively affected the sensitivity of detection. In particular, the H1 assay was not sensitive enough to ensure proper detection of H1 viruses in clinical samples of swine and human origin. Other, more sensitive assays have been published that should be used for those species instead [37]. Unlike H1 viruses, H3 viruses were detected more efficiently also from swine and humans; this is likely due to the use of three multiplexed RT-qPCRs targeting a larger spectrum of H3 sequences. The re-designed N1 assay was able to pick up the N1 subtype from the swine and human origin samples.

## 5. Conclusions

RITA-2 constitutes an important technical update and improved development compared to RITA-1. It also provides functional progress through the combination of RT-qPCR for differential diagnoses of AIV infections; this serves needs to disentangle complex mixtures of viral co-pathogens (NDV, IBV) synergistically acting in respiratory disease of poultry. It is clear that the assay compositions arrayed here will remain subjects to change. AIV in general and Gs/GD viruses, in particular, remain a highly mobile and moving target for sequence-based diagnostic tools. Continuous reference to updated sequence databases and appropriate adaptation of primers and probes are inevitable permanent tasks. An additional functional step forward compared to RITA-1 is provided by the combination of selected RT-qPCR assays with pathotyping RT-qPCR so as to serve the needs of rapid diagnosis of HA and NA subtypes and of the pathotype in HPAI epidemics with a temporal and/or geographic restriction.

## Figures and Tables

**Table 1:** Collection of influenza A virus strains of different host origin and differing subtypes (hemagglutinin, HA, and neuraminidase, NA) used for evaluation of real-time RT-PCRs.

Subtype	Number of Samples	Host				
		Avian	Human	Porcine	Equine	Unknown
**H1**	63	10	17	32		4
**H2**	20	19	1			
**H3**	52	25	11	15	1	
**H4**	20	20				
**H5**	53	53				
**H6**	67	67				
**H7**	42	40			1	1
**H8**	5	5				
**H9**	43	29				14
**H10**	32	30		2		
**H11**	14	12			1	1
**H12**	1	1				
**H13**	10	10				
**H14**	1	1				
**H15**	1	1				
**H16**	4	4				
**HA, total**	**428**	**327**	**29**	**49**	**3**	**20**
**N1**	144	93	17	30		4
**N2**	125	88	11	12		14
**N3**	29	29				
**N4**	10	10				
**N5**	6	5				1
**N6**	28	23		4		1
**N7**	38	34		3	1	
**N8**	37	36			1	
**N9**	11	9	1		1	
**NA, total**	**428**	**327**	**29**	**49**	**3**	**20**

**Table 3:** Reaction volumes used for individual and arrayed RT-qPCRs.

RNA/Mastermix AgPath-ID™ One-Step RT-PCR	Single Reaction	24 Reactions (1 Sample)	96 Reactions (4 Samples)
	1×	26×	100×
RNase free water	2.25 µL	58.5 µL	225 µL
2× RT-PCR Buffer	6.25 µL	162.5 µL	625 µL
RT-PCR Enzyme Mix	0.5 µL	13 µL	50 µL
Primer-Probe mix ^1^	1 µL	26 µL	100 µL
Sample RNA	2.5 µL	65 µL	2.5 µL/well
**Total volume**	**12.5 µL**	**299 µL**	**1000 µL**
Template	2.5 µL/well	2.5 µL/well	2.5 µL/well

^1^ Primer-probe mixes had already been pipetted into plates at fixed positions as shown in Figure 1.

**Table 4:** Identity of virus isolates used as positive controls for RITA-2 batch evaluations.

Positive Control	Subtype	Strain	Cq Value/Reaction
**PTC-1**	H1N1	A/Mallard/Germany/R193/09	23–25
	H5N6	A/White stork/Germany/AR251/2018	21–23
	H9N2	A/Chicken/Egypt/AR538/2017	22–25
	H13N8	A/Larus ridibundus/Germany/R2064/2006	24–26
	IBV-1	AI20298/2019	23–25
**PTC-2**	H2N3	A/Mallard/Germany/Wv677/04	23.65–25
	H6N2	A/Turkey/Mass/3740/65	22–24
	H10N7	A/Mallard/Germany/1490/09	22–24
	H14N5	A/Mallard/Gurjev/263/82	26–27
	IBV	AI20298/2019	23–25
**PTC-3**	H3N8	A/Mallard/Germany/R1648/07	23–25
	H7N7	A/Greylag goose/Germany/AR942/2015	22–24
	H11N9	A/Mallard/Föhr/Wv1499-1503/03	22–24
	H15N9	A/Shearwater/West Australia/2576/79	22–24
	NDV-1	ND/Lentogenic/713/2016	22–24
**PTC-4**	H4N6	A/Mallard/Germany/R485/3/08	21–23
	H8N4	A/Anas latyrhynchos/Germany/R2167/2009	22–24
	H12N5	A/Duck/Alberta/60/76	21–23
	H16N3	A/Herring gull/Germany/R2788/06	23–25
	NDV-2	ND/Velogenic	22–24

**Table 5:** Comparison of the results of clinical samples obtained with RITA-2 and by sequencing or subtyping with other RT-PCRs. Results for sample originating from Germany are shown in Table S2.

Country	Species	No. of Farms	RITA-2	Subtyping/ Other PCRs	Sequencing
Egypt	Chicken	2	H5, N8	H5, N8	H5 HP, N8 [22]
Egypt	Turkey	2	H5, N8	H5, N8	H5 HP, N8 [22]
Egypt	Ducks	1	H5, N8	H5, N8	H5 HP, N8
Egypt	Ducks	7	H5, N8	H5, N8	H5 HP
Egypt	Chicken	3	H9, N2	H9, N2	H9, N2 [22]
Egypt	Chicken	2	H5, N1	H5, N1	H5, N1 [22]
Egypt	Duck	5	H5, N8	H5, N8	H5 HP
Egypt	Chicken	5	H5, H9, N8, N2	H5, H9, N2	H5 HP [22]
Egypt	Chicken	3	H5, N2	H5, N2	H5 HP, N2 [22]
Egypt	Chicken	2	H5, H9, N8, N2	H5, H9, N8, N2	H5, H9, N8, N2 [10]
Egypt	Chicken	1	H5, H9, N8, N2, IBV, NDV	H5, H9, N8, N2, IBV, NDV	H5, H9, N8, N2, IBV [10]
Egypt	Chicken	1	H5, N8, IBV, NDV	H5, N8, IBV, NDV	H5, N8, IBV [10]
Bangladesh	Duck	13	H4, N6	H4, N6	H4, N6 [23]

## Data Availability

All data pertinent to this study are presented in tables and figures in the main text or in the Supplementary Materials.

## Supplementary Materials

The following supporting information can be downloaded at: https://www.mdpi.com/article/10.3390/v14020415/s1, Table S1. Identity of Infectious Bronchitis and Newcastle Disease virus isolates used to validate specific RT-qPCRs; Table S2. Analysis of clinical samples obtained during active or passive monitoring of wild birds and poultry in Germany, 2020–21. Table S3. Summary statistics of intra- and interassay variations of the RITA-2 array; Table S4. Raw data of inter- and intra-assay variations.
