# Supplementary material for: Improved Subtyping of Avian Influenza Viruses Using an RT-qPCR-Based Low Density Array: ‘Riems Influenza a Typing Array’, Version 2 (RITA-2)
Source: Viruses. 2022 Feb 17;14(2):415. doi: 10.3390/v14020415 (PMC8879595; doi:10.3390/v14020415)
Supplement: Supplementary file 1 [file viruses-14-00415-s001.zip › viruses-1554791-supplementary.pdf]

Table S1. Identity of Infectious Bronchitis and Newcastle Disease virus isolates used to validate specific RT-qPCRs.

Virus species	Strain	Patho-/Genotype
ND	ND/713/2016	Lasota vaccine/Lentogenic, 2
	A/Chicken/Egypt/AR518/2017	Lentogenic, 2
	A/Chicken/Egypt/AR593/2018	Lentogenic, 2
	ND/NR730/2016	Velogenic, 7b
	A/Chicken/Egypt/AR551/2018	Velogenic, 7b
	A/Chicken/Egypt/AR563/2018	Velogenic, 7b
	A/Chicken/Egypt/AR589/2018	Velogenic, 7b
	A/Chicken/Egypt/AR549/2018	Velogenic, 7b
IBV	AI20298/2019	4/91 IBV vaccine/793B
	A/Chicken/Egypt/AR545/2018	Egyptian variant 2
	A/Chicken/Egypt/AR63/2018	Egyptian variant 2
	A/Chicken/Egypt/AR593/2018	Egyptian variant 2
	A/Chicken/Egypt/AI20290/2019	Egyptian variant 2
	A/Chicken/Egypt/ AI20293/2019	Egyptian variant 2
	A/Chicken/Egypt/ AI20297/2019	Egyptian variant 2

Table S2. Analysis of clincial samples obtained during active or passive monitoring of wild birds and poultry in Germany, 2020-21.

Sample id.	Species	Subtype[1]	Pathotype	H5-8	H7-8	RITA-2	Cq
2020AI01301	Mallard	H1N1		HxN1	HxN1	H1N1	26.46
2020AI01399	Mallard	H1N2		HxN2	HxNx	H1N2	30.62
2020AI01045	Mallard	H2N5		HxNx	HxNx	H2N5	35.53
2020AI01071	Mallard	H3N8		HxN8	HxNx	H3N8	31.13
2020AI00046	Tufted duck	H5N1	LP	H5LPN1	nt	H5N1	25
2020AI03671	Greyleg goose	H5N1	HP	H5N1	nt	H5N1	33.05
2021AI01598	Greyleg goose	H5N1	HP	H5(HP, 2344b)N1	nt	H5N1	32.36
2021AI01602	Chicken	H5N1	HP	H5(HP, 2344b)N1	nt	H5N1	15.69
2021AI01605	Barnacle goose	H5N1	HP	H5(HP, 2344b)N1	nt	H5N1	26.81
2021AI03752	Barnacle goose	H5N1	HP	H5(HP, 2344b)N1	nt	H5N1	24.16
2021AI03933	Chicken	H5N1	HP	H5(HP, 2344b)N1	nt	H5N1	18.24
2021AI03934	Chicken	H5N1	HP	H5(HP, 2344b)N1	nt	H5N1	20.99
2020AI01397	Mallard	H5N2	LP	H5(LP)N2	nt	H5N2	29,07
2020AI01462	Mute swan	H5N3	LP	H5LPN3	nt	H5N3	31.75
2020AI03422	Red knot	H5N3	HP	H5(HP, 2344b)N1	nt	H5N3	28.23
2021AI00094	Red knot	H5N3	HP	H5	nt	H5N3	32.63
2021AI00382	Common buzzard	H5N3	HP	H5(HP, 2344b)N3	nt	H5N3	25,09
2021AI00394	Red knot	H5N3	HP	H5(HP, 2344b)N3	nt	H5N3	26,05
2021AI01068	Red knot	H5N3	HP	H5(HP, 2344b)N3	nt	H5N3	22.90
2021AI01736	Peregrine falcon	H5N3	HP	H5(HP, 2344b)N3	nt	H5N3	23,12
2021AI01497	Gull	H5N4	HP	H5(HP, 2344b)Nx[2]	nt	H5N4	33.05
2021AI01498	Mute swan	H5N4	HP	H5(HP, 2344b)Nx[2]	nt	H5N4	27,05
2021AI01743	Tufted duck	H5N4	HP	H5(HP, 2344b)Nx[2]	nt	H5N4	35.93
2021AI01766	Peregrine falcon	H5N4	HP	H5(HP, 2344b)Nx[2]	nt	H5N4	33.76
2020AI02166	Common buzzard	H5N5	HP	H5(HP, 2344b)Nx[2]	nt	H5N5	16.28
2020AI02246	Eurasian wigeon	H5N5	HP	H5(HP, 2344b)Nx[2]	nt	H5N5	31.59
2020AI02255	Bean goose	H5N5	HP	H5(HP, 2344b)Nx[2]	nt	H5N5	21.75
2020AI02419	Chicken	H5N5	HP	H5(HP, 2344b)Nx[2]	nt	H5N5	28,05
2020AI02170	White-tailed sea eagle	H5N5/N8	HP	H5(HP, 2344b)N8	nt	H5N5/N8	27,04
2020AI02196	Barnacle goose	H5N5/N8	HP	H5(HP, 2344b)N8	nt	H5N5/N8	20.89
2020AI00326	Turkey	H5N8	HP	H5(HP, 2344b)N8	nt	H5N8	21.20
2020AI00350	Turkey	H5N8	HP	H5(HP, 2344b)N8	nt	H5N8	23.14
2020AI02315	Curlew	H5N8	HP	H5(HP, 2344b)N8	nt	H5N8	19.93
2020AI03043	Crescent sandpiper	H5N8	HP	H5(HP2344b)N8	nt	H5N8	24.78
2020AI03056	Mallard	H5N8	HP	H5(HP, 2344b)N8	nt	H5N8	24.57
2020AI03433	Turkey	H5N8	HP	H5(HP, 2344b)N8	nt	H5N8	21.35
2021AI00092	Barnacle goose	H5N8	HP	H5(HP, 2344b)N8	nt	H5N8	21.66
2021AI00383	Greyleg goose	H5N8	HP	H5(HP, 2344b)N8	nt	H5N8	22,98
2021AI00385	Laughing gull	H5N8	HP	H5(HP, 2344b)N8	nt	H5N8	25.63
2021AI00386	Kestrel	H5N8	HP	H5(HP, 2344b)N8	nt	H5N8	22.98
2021AI01495	Barnacle goose	H5N8	HP	H5(HP, 2344b)N8	nt	H5N8	27.32
2021AI01737	Peregrine falcon	H5N8	HP	H5(HP, 2344b)N8/N3	nt	H5N8	30.87
2021AI01742	Eagle owl	H5N8	HP	H5(HP, 2344b)N8	nt	H5N8	28.60
2021AI01752	Great cormorant	H5N8	HP	H5(HP, 2344b)N8	nt	H5N8	32.06
2021AI02466	Mute swan	H5N8	HP	H5(HP, 2344b)N8	nt	H5N8	20.78
2020AI02379	Barnacle goose	H5N8/N5	HP	H5(HP, 2344b)N8	nt	H5N8/N5	24.93
2020AI02279	Eurasian wigeon	H5Nx	HP	H5(HP, 2344b)N8	nt	H5Nx	30,55
2021AI00432	Turkey	H6N1		HxN1	HxN1	H6N1	27.88
2021AI02559	Dwarf chicken	H7N3	LP	nt	LP H7N3	nt	28.32
2021AI02560	Dwarf chicken	H7N3	LP	nt	LP H7N3	nt	28.52
2021AI02561	Dwarf chicken	H7N3	LP	nt	LP H7N3	H7N3	28.52
2021AI03100	Domestic duck	H7N3	LP	nt	LP H7N3	H7N3	32.69
2020AI03226	Mallard	H7N7	LP	nt	LP H7N7	H7N7	27.70
2020AI02242	Mallard	H8N4		nt	HxNx	H8N4	32.58
2020AI01400	Mallard	H9N1		HxN1	nt	H9N1	29.42
2020AI01047	Mute swan	H9N8		HxN8	HxNx	H9N8	32.23
2020AI01693	Mallard	H10N6		HxN6	HxNx	H10N6	29.14
2020AI01055	Mallard	H11(?)N9		nt	HxN9	H11N9	29.54
2020AI01469	Mute swan	H11N1		HxN1	HxN1	H11N1	24.48
2020AI03651	Mallard	H12N2		HxN2	HxNx	H12N2	32.30
2020AI01401	Weißohrturako	H12N5		HxN5	HxNx	H12N5	25.39
2020AI02736	Herring gull	H13N2		HxN2	nt	H13N2	23.84

[1] – Subtype determined according to single RT-qPCRs and/or partial HA/NA sequencing.

2 – N4 and N5 RT-qPCRs had not been included in the H5-8 array when these samples were tested.

nt – not tested.

Cq – threshold value obtained with the generic M-RTqPCR included in the RITA-2 assay

Table S3. Summary statistics of intra- and interassay variations of the RITA-2 array.

Subtype	Intra-assay variation				Inter-assay variation		
	Day one		Day two				
	SD	CV%	SD	CV%	Mean	SD	CV%
H1	0.007	0.036	0.007	0.036	19.47	0.023	0.122
H2	0.007	0.035	0.007	0.035	19.69	0.018	0.092
H3	0.014	0.082	0.028	0.164	17.17	0.044	0.258
H4	0.007	0.037	0.056	0.303	18.65	0.034	0.182
H5	0.007	0.021	0.014	0.043	32.64	0.012	0.038
H6	0.007	0.024	0.098	0.344	28.67	0.075	0.263
H7	0.007	0.049	0.007	0.049	14.24	0.058	0.407
H8	0.035	0.149	0.056	0.238	23.69	0.046	0.196
H9	0.007	0.038	0.014	0.077	18.30	0.009	0.052
H10	0.070	0.361	0.007	0.036	19.54	0.045	0.234
H11	0.021	0.114	0.014	0.076	18.49	0.015	0.081
H12	0.014	0.082	0.028	0.165	17.08	0.021	0.126
H13	0.014	0.072	0.056	0.291	19.39	0.033	0.173
H14	0.007	0.036	0.007	0.036	19.17	0.023	0.124
H15	0.007	0.025	0.028	0.102	27.62	0.035	0.130
H16	0.014	0.082	0.021	0.124	17.09	0.020	0.120
N1	0.069	0.319	0.014	0.065	21.62	0.068	0.318
N2	0.056	0.261	0.014	0.065	21.60	0.033	0.155
N3	0.021	0.108	0.042	0.216	19.60	0.046	0.236
N4	0.042	0.228	0.007	0.038	18.59	0.026	0.141
N5	0.049	0.264	0.021	0.113	18.68	0.046	0.249
N6	0.028	0.151	0.070	0.380	18.63	0.059	0.320
N7	0.056	0.390	0.063	0.439	14.47	0.051	0.353
N8	0.035	0.174	0.028	0.140	20.18	0.033	0.163
N9	0.021	0.124	0.056	0.331	17.07	0.040	0.236
ND	0.007	0.028	0.021	0.086	24.43	0.042	0.173
IBV	0.077	0.295	0.028	0.107	26.27	0.072	0.276

Table S4. Raw data of inter- and intra-assay variations.

Subtype	Day one			Day two		
	Cq value	Cq value	Mean	Cq value	Cq value	Mean
H1	19.45	19.46	19.45	19.50	19.49	19.49
H2	19.70	19.71	19.70	19.68	19.67	19.67
H3	17.13	17.15	17.14	17.23	17.19	17.21
H4	18.65	18.64	18.64	18.70	18.62	18.66
H5	32.65	32.66	32.65	32.63	32.65	32.64
H6	28.64	28.63	28.63	28.79	28.65	28.72
H7	14.30	14.29	14.29	14.20	14.19	14.19
H8	23.65	23.70	23.67	23.76	23.68	23.72
H9	18.31	18.30	18.30	18.29	18.31	18.3
H10	19.61	19.51	19.56	19.53	19.52	19.52
H11	18.51	18.48	18.49	18.50	18.48	18.49
H12	17.08	17.10	17.09	17.09	17.05	17.07
H13	19.38	19.40	19.39	19.35	19.43	19.39
H14	19.19	19.20	19.19	19.15	19.16	19.15
H15	27.65	27.66	27.65	27.62	27.58	27.6
H16	17.10	17.12	17.11	17.10	17.07	17.08
N1	21.62	21.72	21.67	21.59	21.57	21.58
N2	21.56	21.64	21.60	21.59	21.61	21.6
N3	19.62	19.65	19.63	19.54	19.60	19.57
N4	18.57	18.63	18.60	18.58	18.59	18.58
N5	18.75	18.68	18.71	18.64	18.67	18.65
N6	18.69	18.65	18.67	18.65	18.55	18.6
N7	14.53	14.45	14.49	14.51	14.42	14.46
N8	20.23	20.18	20.20	20.15	20.19	20.17
N9	17.11	17.08	17.09	17.10	17.02	17.06
ND	24.40	24.39	24.39	24.45	24.48	24.46
IBV	26.38	26.27	26.32	26.25	26.21	26.23
